# Efficacy of positive airway pressure therapy and high flow nasal cannula oxygen in acute cardiogenic pulmonary oedema: A protocol for systematic review and network meta-analysis

**DOI:** 10.3389/fmed.2022.992491

**Published:** 2022-12-07

**Authors:** Jianyi Niu, Zhenfeng He, Shanshan Zha, Qiaoyun Huang, Wei Fu, Shengchuan Feng, Lili Guan, Luqian Zhou, Rongchang Chen

**Affiliations:** ^1^State Key Laboratory of Respiratory Disease, National Clinical Research Center for Respiratory Disease, Guangzhou Institute of Respiratory Health, First Affiliated Hospital of Guangzhou Medical University, Guangzhou, China; ^2^Respiratory Mechanics Laboratory, Guangzhou Institute of Respiratory Health, First Affiliated Hospital of Guangzhou Medical University, Guangzhou, China; ^3^Key Laboratory of Shenzhen Respiratory Diseases, Institute of Shenzhen Respiratory Diseases, Shenzhen People's Hospital (The First Affiliated Hospital of Southern University of Science and Technology, The Second Clinical Medical College of Jinan University), Shenzhen, China

**Keywords:** acute cardiogenic pulmonary edema, non-invasive ventilation, high-flow nasal cannula oxygen, Bayesian meta-analysis, protocol

## Abstract

**Introduction:**

Positive airway pressure (PAP) therapy is currently the first-line respiratory support technique for acute respiratory failure (ARF) due to acute cardiogenic pulmonary edema (ACPE), but the accompanied adverse events and patient's intolerance with treatment in some cases limited its use in clinical practice. Some recent trials indicated that high-flow nasal cannula oxygen (HFNO) is a promising alternative to PAP therapy. In order to choose the optimum treatment for patients with ACPE, this network meta-analysis will firstly compares the efficacy of HFNO, PAP, and conventional oxygen therapy (COT).

**Methods and analysis:**

The Preferred Reporting Items for Systematic Review and Meta-Analysis Protocols 2015 statement and its extension for network meta-analysis will be followed in the conduct of this investigation. We will examine these databases: PubMed, EMBASE, Cochrane Central Register of Controlled Trials and Web of Science. The ClinicalTrials.gov and World Health Organization International Clinical Trials Registry Platform Search Portal will be used to search ongoing trials. Only randomized controlled trials meeting the eligibility criteria will be included. Through the Cochrane Collaboration's tool, the included studies' risk of bias will be assessed. The pairwise meta-analysis will be performed with RevMan 5.4.1 software. A Bayesian network meta-analysis will use random-effects models to derive odds ratios for the treatment effects of all interventions compared to each other using R software (version 3.6.1), and the rjags and gemtc packages. The Q statistic and I^2^ index will be used for investigating the heterogeneity, and subgroup analysis or sensitivity analysis will be used to explore the source of heterogeneity. In addition, the Grading of Recommendations Assessment, Development and Evaluation system will be used to inspect the quality of evidence.

## Introduction

Positive airway pressure (PAP) therapy is currently the first-line respiratory support technique for patients with acute respiratory failure (ARF) due to acute cardiog*e*nic pulmonary edema (ACPE) (1). Compared to conventional oxygen therapy (COT), it was discovered that PAP could successfully reduce the in-hospital intubation rate and death in such patients (2). However, previous studies have found that PAP in ACPE patients has an increased risk of adverse events, including hypotension, acute myocardial infarction, aggravated right heart failure, aggravated hypercapnia, aspiration pneumonia, pneumothorax, etc. (3, 4). Moreover, relevative lower patient's tolerance, the demand for well-trained PAP operators and well-established monitoring procedures restrict its clinical practice.

High-flow nasal cannula oxygen (HFNO), a novel respiratory support method with greater comfort than PAP, can deliver heated and humidified air-oxygen mixture at high flow rates (up to 60 L/min) to flush the dead space in the upper airway, and therefore reduce the rebreathed carbon dioxide. Additionally, it can create low-level (about 3 cmH_2_O) positive airway pressure (5). A recent randomized controlled trial (RCT) involving 128 patients with ACPE found that after 1 h of treatment, HFNO could significantly improve breathing rate compared to COT (6). Moreover, HFNO was reported to have more ventilator free-days and lower mortality rate than PAP among patients with ARF (7), and therefore is a promising non-invasive support approach for patients with ACPE (8, 9).

For now, there is a lack of high-quality evidence of the efficacy of HFNO and PAP in treating patient with ACPE-related ARF due to limited sample size in previous clinical trials (10–13) and no suffient data to distinguish the better one from PAP and HFNO in the treatment of ACPE-related ARF. By using network meta-analysis, not only direct comparison of efficacy differences between the two methods of respiratory support, but also further comprehensive analysis through indirect comparison based on logical inference.

## Objective

This systematic literature review and network meta-analysis will aim to comprehensively compare the efficacy of PAP and HFNO in patients with ACPE by direct and indirect comparisons. Specifically, what are the efficacy of PAP and HFNO in patients with ACPE as reported in the research published from their inception to 30 June 2022. The primary outcome will be hospital mortality. Important short-term secondary outcomes will be included: short-term changes in respiratory-related indicators such as respiratory rate; and endotracheal intubation, treatment intolerance, length of hospital stay and intensive care unit stay and incidence of adverse events related to non-invasive respiratory support.

## Methods

This protocol has been written in accordance with the Preferred Reporting Items for Systematic Review and Meta-Analysis Protocols 2015 statement (PRISMA-P) and its extension for network meta-analysis (14, 15). This protocol has also been registered in the International Prospective Register of Systematic Reviews (PROSPERO, CRD42022343499).

### Eligibility criteria

#### Study designs

Only RCTs will be included. We will include RCTs that analyzed pairwise comparisons of NPPV, HFNO and COT in patients with ACPE. We will also include studies that reported full-text and unpublished data, but we will exclude case report studies and conference articles.

#### Participants

RCTs enrolling adults older than 18 years old who were diagnosed with ACPE (16) and were treated with at least any of the two modalities of PAP therapy, HFNC, and COT and compared will be included. ACPE is diagnosed by the presence of the following clinical symptoms: sudden onset of respiratory distress or failure, signs of dyspnea, possible orthopnea, engorgement of the neck vessels; by chest radiograph, electrocardiogram, serum biomarkers, or ultrasound cardiogram and other examinations to assist diagnosis. Patients with acute respiratory failure due to other heart and lung diseases will be excluded. PAP therapy used for purposes such as assisting weaning will also be excluded.

#### Interventions

All interventions considered in this network meta-analysis were non-invasive respiratory support strategies (PAP vs. HFNO, PAP vs. COT or HFNO vs. COT). PAP therapy will provide positive pressure ventilation through a non-invasive interface (mask or helmet), and ventilation modes include: continuous positive airway pressure and bilevel positive airway pressure. HFNO will deliver a high flow rate (up to 60 L/min) of a heated and humidified air-oxygen mixture through a nasal cannula. And COT will deliver low-flow oxygen through a traditional nasal cannula or a venturi system mask.

#### Outcomes

The primary outcome of this study will be all-cause mortality, defined as the longest available in the first 30 days after randomization. And Important short-term secondary outcomes will include: short-term changes in respiratory-related indicators such as respiratory rate, PaO_2_/FiO_2_, SpO_2_ and arterial blood gas results (PaO_2_, PaCO_2_ and PH) 1 h post-intervention; and long-term secondary outcomes: endotracheal intubation, treatment intolerance, treatment failure (the combination of all-cause mortality, endotracheal intubation and intolerance to the allocated treatment), length of hospital stay and intensive care unit stay and incidence of adverse events related to non-invasive supportive care.

### Information sources and search strategy

#### Electronic searches

Four databases: PubMed, EMBASE, Web of Science and Cochrane Central Register of Controlled Trials (CENTRAL) will be searched from their inception to 30 June 2022. The literature will be limited to English, but there will be no restrictions on publication. We will search again before the final analysis.

#### Searching other resources

We will search for unfished studies through ClinicalTrials.gov and the World Health Organization International Clinical Trials Registry Platform Search Portal (http://apps.who.int/trialsearch/). We will contact the authors for further research information when necessary. In order to ensure that the search is comprehensive, we will look through the reference of the included studies or reviews to identify the relevant reports.

### Study records

#### Data management

All previously searched literature will be imported into EndNote Version X9.1 software (Clarivate Analytics, Philadelphia, PA, USA). Duplicate inclusions will be screened out and deleted. Titles and abstracts of imported articles will be independently checked by two investigators (JN and ZH), and articles will be downloaded if met the eligibility criteria, or if there is any uncertainty about the inclusion of a particular study.

#### Selection process

Two principal investigators (JN and ZH) will independently read important articles and decide whether to include them according to the inclusion criteria, and if there is a disagreement between the two investigators, a third investigator (LG) will provide comments and decide whether to include them or not finally. The reasons for studies not being included will also be explained in detail.

#### Data collection process

The data will be mainly divided into two categories: dichotomous outcomes and continuous outcomes, which will be independently extracted from the studies determined to be included by two researchers (JN and ZH) in a standard format. The following study data will be extracted: (1) basic characteristics of studys, such as first author, year of publication, journal and study design; (2) basic characteristics of patient enrollment, including number of patients in each group, age, gender, PaO_2_/FiO_2_, PaCO_2_, cardiac function and disease severity at the starting point of PAP/HFNC/COT; (3) PAP therapy characteristics, such as mode of PAP, parameter settings, the type of interface used for positive pressure therapy and location; (4) primary and secondary outcomes. We will only retrieve data from the first stage of randomized crossover studies due to carry-over effects. In the absence of a reasonable washout period, the first-stage intervention will have an impact on the second-stage outcome. After the extraction process is complete, we will compare the two extracted data. If a disagreement is found, the two independent investigators will first try to reach an consensus through discussion. But if differences still exist after the discussion, the third investigator (LG) will participate in the discussion to help draw a final conclusion.

### Risk of bias in individual studies

The Cochrane Collaboration's tool, a classic RCTs quality assessment tool, will be used to assess the risk of bias of included studies (17, 18), and will also be done independently by two investigators (JN and ZH). The following aspects will be assessed: (1) random sequence generation (selection bias), (2) allocation concealment (selection bias), (3) blinding of participants and personnel (performance bias), (4) blinding of outcome assessment (detection bias), (5) incomplete outcome data, (6) selective reporting (reporting bias), and (7) other sources of bias. Each area will be judged as three levels: “low risk,” “uncertain risk” or “high risk” by investigator. Support for these judgments will be further clarified in the risk of bias table. Disagreements will first be dealt with after discussion between two investigators, and a third investigator (LG) will provide advice when agreement can not be reached.

### NMA relevant assumptions

Compared with pairwise meta-analyses, network meta-analyses require stricter methodological and statistical assumptions, including similarity, transitivity, and consistency. Similarity is a qualitative assessment of each article in terms of methodology, specifically the clinical characteristics of the study subjects, treatment interventions, and outcomes measures. Consistency and transitivity have certain similarities, both of which are assessments of differences between direct and indirect comparisons, but they are used from statistical and logical perspectives, respectively. Two investigators (JN and ZH) will independently complete the above three assessments, and in the event of disagreement, the other (LG) will provide advice.

### Data synthesis

Analyses will be done primarily using RevMan software (version 5.4.1), R software (version 3.6.1), and the rjags and gemtc packages.

We will use the Review Manager V.5.4.1 software (Cochrane Collaboration, Oxford, UK) for pairwise meta-analysis to calculate odds ratio and its 95% confidence interval for dichotomous outcomes, and the standardized mean differences and 95% confidence for continuous outcomes. Heterogeneity will be assessed by the Cochran Q statistic and the I^2^ statistic.

We will perform a series of pairwise Bayesian meta-analyses using random-effects models, followed by network meta-analyses using a Bayesian framework, to derive odds ratios for the treatment effects of all interventions compared to each other by using R software (version 3.6.1). For mortality, the probability that each preventive strategies to be the best among the preventive strategies will be determined by evaluating the rank probabilities. Based on the available clinical evidence, the higher the probability that an intervention will be ranked 1, the higher the probability that it will be the best intervention. We will estimate the inconsistency between direct and indirect comparisons by using the node-splitting approach.

#### Measures of treatment effect

Dichotomous outcomes such as patient mortality and the incidence of endotracheal intubation will be described using the odds ratio with 95% confidence intervals. For continuous outcomes such as length of hospital stay, changes in respiratory rate, etc., the standardized mean difference with 95% confidence intervals will be used. When standardized mean differences are not reported, we will calculate them based on other message reported in the study, for example, t-statistics or *p*-values, according to Altman and Bland (19).

#### Dealing with missing data

We will email the original authors when data are missing or irrelevant, or we will utilize a technique to transform them into usable data (18, 20, 21). We will adhere to the guidelines of the Cochrane Handbook for Systematic Reviews of Intervention (16) if the missing data can not be obtained: (1) make precise the hypothesis of any methods used to handle the missing data; (2) sensitivity analyses will be used to assess how sensitive results are to reasonable changes in the assumptions that are made; (3) consider the potential effect of the missing data as limitations in our study.

#### Assessment of heterogeneity

Using the Q statistic and I^2^ index, we will examine statistical heterogeneity among the included research. Heterogeneity will be regarded as statistically significant if the Q statistic's *p* < 0.10. Low, moderate or high heterogeneity is judged by I^2^ value <25%, 25–50%, or >50%, respectively. I^2^ values >50% will be regarded as substantial heterogeneity. We will apply a fixed-effect model if I^2^ values ≤ 50%. We will attempt to explain the source of heterogeneity by subgroup analysis, sensitivity analysis, or the use of a random-effects model when significant heterogeneity (I^2^ > 50%) is discovered. If significant heterogeneity can not be adequately explained, descriptive and qualitative summaries will be provided instead of meta-analysis (18).

#### Additional analyses

Possible sources of heterogeneity will be determined by subgroup analysis based on the presence or absence of hypercapnia (PaCO_2_ ≥ 45 mmHg), basline oxygenation index (PaO_2_/FIO_2_) < 200, disease severity at the starting point of PAP/HFNC/COT, mode of PAP therapy (continuous positive airway pressure or bilevel positive airway pressure), the type of interface used for positive pressure therapy, location of therapy (intensive care unit vs. emergency room) and the left ventricular ejection fraction <50%. Sensitivity analyses will also be performed to detect sources of heterogeneity. When significant heterogeneity is present, we will identify the source of the apparent heterogeneity by exploring and comparing the effect of that study on the pooled estimates by excluding different studies in turn. After successfully identifying the source of heterogeneity, we will need to review the literature again to assess its quality and bias, discuss its reasons for apparent heterogeneity, and ultimately decide whether to keep or exclude the particular study. If once the study is excluded, the reason for its removal will be explained in the discussion. If quantitative synthesis is not appropriate, a systematic review will be used. This approach will present information in the form of text or tables to explain and summarize the characteristics and results of the included studies in detail.

### Assessment of publication bias

When the final number of included studies is ≥10, we will assess publication bias by means of a funnel plot. In addition, in order to assess the reporting bias, we will search the database of the registered protocols mentioned above to confirm that whether the study's prespecified outcomes have not been reported.

### Confidence in cumulative estimate

The investigators will use the Grading of Recommendations Assessment, Development and Evaluation (GRADE) system to rate the quality of evidence for all outcomes included in the study. In this system, four quality grades (high, moderate, low and very low) will be used to evaluate the quality of the evidence. When evaluating RCTs, the quality of the evidence will be reduced when there are study limitations, inconsistent results, indirectness of the evidence, imprecision, or reporting bias, as RCTs are often considered to be of high quality (22, 23). Estimates of network effects for each potential comparison and each outcome, as well as GRADE ratings for the certainty of evidence, will be described in the summary of results table.

## Discussion

Until now, the efficacy of HFNO and PAP for treating ARF caused by ACPE is unclear, and a further meta-analysis comparing the effectiveness of these two methods is needed. As only a few studies directly evaluate the clinical efficacy of PAP and HFNO in treating ACPE, we therefore employ a network meta-analysis to obtain more evidence by indirectly comparing PAP and HFNO.

Continuous positive airway pressure and bilevel positive airway pressure are the most frequently utilized PAP modes in clinical settings to treat ACPE. They can decrease work of breathing and enhance lung compliance, gas exchange, and cardiac output by decreasing left ventricular afterload, accelerating alveolar fluid clearance, improving pulmonary compliance and ventilation/perfusion (24). However, a certain risk of adverse effects still exists when receiving PAP. For example, PAP would worsen right heart failure by increasing right ventricular afterload and leading to hypotension (25). Additionally, PAP should be operated by the well-trained medical staff, which may limit its application in some clinical situations.

HFNO has become an emerging non-invasive respiratory support technique in recent years. It could reduce carbon dioxide rebreathing by flushing respiratory dead space and generate a positive end-expiratory pressure effect. In addition, it is also easy to operate and better tolerated than PAP. However, the positive end-expiratory pressure level is relatively low, unstable, uncontrollable and affected by many factors (26). Moreover, its clinical effectiveness in ACPE has not yet been proved.

In this meta-analysis, we hope to provide more evidence on the most appropriate form of respiratory support methods for the treatment of cardiogenic pulmonary edema and to explore the additional benefit among different clinical condition (with hypercapnia, baseline oxygenation index < 200, different disease severity at the starting point of PAP/HFNC/COT, use of different PAP modes, use of different type of interface, different treatment location, and left ventricular ejection fraction <50%).

## Author contributions

JN, ZH, SZ, and QH were involved in the idea and design of the study. They also helped to draft the manuscript that was submitted and critically review it for significant intellectual content. The search plan was created with input from QH and WF. SZ and ZH handled the data management and selection procedures. JN and SF were in charge of gathering the data. JN and WF contributed to the evaluation of the risk of bias. All authors were involved in the data analysis, revisions to the paper, overall responsibility for the work, and approval of the manuscript's final draft.
